# Retraction: TUSC3 induces autophagy in human non-small cell lung cancer cells through Wnt/β-catenin signaling

**DOI:** 10.18632/oncotarget.28315

**Published:** 2022-12-06

**Authors:** Yun Peng, Jun Cao, Xiao-Yi Yao, Jian-Xin Wang, Mei-Zuo Zhong, Ping-Ping Gan, Jian-Huang Li

**Affiliations:** ^1^International Medical Center, Xiangya Hospital, Central South University, Changsha 410008, P.R. China; ^2^Department of Medical Oncology, The Affiliated Cancer Hospital of Xiangya School of Medicine, Central South University, Changsha 410008, P.R. China; ^3^Department of Oncology, Xiangya Hospital, Central South University, Changsha 410008, P.R. China; ^4^School of Information Science and Engineering, Central South University, Changsha 410008, P.R. China

**This article has been retracted:** Oncotarget requested the original data for this paper to address concerns regarding image duplication. The authors were unable to retrieve the data. As a result, all authors have agreed to the retraction of this paper from Oncotarget. The University was notified about the issues with the paper.

Original article: Oncotarget. 2017; 8:52960–52974. 52960-52974. https://doi.org/10.18632/oncotarget.17674

